# A Case of Leprosy—Elephantiasis Græcorum

**Published:** 1879-04-20

**Authors:** 


### *A CASE OF LEPROSY—ELEPHANTIASIS GRÆCORUM.*

REPORTED TO SAN FRANCISCO MEDICAL SOCIETY.

Dr. Charles Blach, City Physician, after reading a paper on the subject, exhibited to the society a Chinaman who presented a well-marked case of leprosy. His name is Yow Gown, age 34, has a wife and three children. Former occupation, merchant. Born in a district of China called Seven Creeks, a low, marshy region. The disease commenced whilst he was in China, attacking both sides of the face at the same time. He had never suffered any injury of the face, nor syphilis. He knew of no special disease in his family. The tumefaction gives no pain unless pressure is applied. General health is good, and he can imagine no cause for the disease. Dr. Blach had conversed with Dr. Loo Chan Foey, an educated Chinese physician residing in San Francisco, and from him procured the following statement.

The name of the disease in Chinese is *Sang Foongler Fat Foong*. It is most common in low, marshy districts, and is caused by miasmata and unwholesome food. It is not considered contagious, except from sleeping or eating with the person affected. Thinks it can be contracted by sexual intercourse. It is most common among the poor. The treatment is not well defined. A decoction of a species of sea-weed is much employed, and some Chinese physicians use arsenic. An ointment is applied containing amber, camphor, cinnabar, nut-oil, spermaceti and musk. No salt food, nor bread made with yeast, nor fowls or game are allowed. The patient is confined to rice and fresh pork.

Dr. Blach further remarked that the disease is described by Duhring as an endemic, chronic, malignant affection, characterized by alterations in the cutaneous nerve and bone tissues, resulting in anesthesia, ulceration, necrosis, general atrophy and deformity. Its course is slow, requiring years for its full development. Authors generally agree that it is not communicable except under peculiar conditions. The treatment laid down by modern authorities is palliative rather than curative, being anti-miasmatic, with abstinence from salt food. According to the *Custom's Gazette*, published at Shanghai, there was admitted at Takow, in a dry district, only one case of leprosy in six months; while at Hamkow, which is a malarial district, ninety-four cases were admitted into the hospital during the same period. The disease might possibly become endemic in some of the interior malarious valleys of California, with an overcrowded population of a low order, living on poor food. But in elevated regions and other localities he did not think it possible.

Dr. Isaac Rivas remarked that leprosy was not rare in Mexico. There was an hospital exclusively appropriated to it in the city of Mexico, which had been in existence eighty years. He did not consider it contagious, as none of the nurses had ever contracted it. He had also had four cases in his own practice, which seemed to confirm that opinion.

Dr. Stivers did not regard it as a contagious disease. He mentioned the case of a woman who died from it in the Almshouse in this city, where she had been employed for some time in washing, etc. There was no communication of disease. The Chinese themselves do not believe in its contagiousness. Dr. S. thinks it is due to malaria and the use of salt and decayed meats.

Dr. H. M. Fiske made mention of a small leper rancheria in Amador county which was supported by voluntary contribution from the Chinese. The Dr. regarded the disease as contagious.

Dr. Ayer inquired if females were as liable to the disease as men.

Dr. J. Regensburger answers that writers say they are not.

Dr. James Blake asked if a leprous mother could communicate it to her child by nursing?

Dr. Loo Chan Foey answered in the affirmative.

Dr. M. Regensburger exhibited pathological specimens, illustrating the following cases:

1. A Mexican woman was beaten by a man with his fists, so as to bruise her face badly. She became delirious, and finally died in the hospital, it was supposed from meningitis caused by violence. But on examination, the brain and membranes were found in a perfect condition. The lungs were somewhat congested, and the right side of the heart was slightly engorged. But no adequate cause of death was discovered.

2. Francis, P., aged 30, baker, had always attended to his work without complaint, and was considered a healthy man. His death was sudden. Dissection revealed hypertrophy of heart, with malignant disease of the organ.

3. Madame R., native of France, had complained for some time of pain in the region of the heart, with asthma. During the day she drank wine and brandy in small quantities, and after retiring early, died suddenly. Cause, hypertrophy of heart, with cartilaginous aortic valves, and some fatty degeneration.—*San Francisco Med. and Surg. Journal.*

